# Novel lithium-nitrogen compounds at ambient and high pressures

**DOI:** 10.1038/srep14204

**Published:** 2015-09-16

**Authors:** Yanqing Shen, Artem R. Oganov, Guangri Qian, Jin Zhang, Huafeng Dong, Qiang Zhu, Zhongxiang Zhou

**Affiliations:** 1 Department of Physics, Harbin Institute of Technology, Harbin 150001, China; 2 Skolkovo Institute of Science and Technology, Skolkovo Innovation Center, 3 Nobel St., Moscow 143026, Russia; 3 Moscow Institute of Physics and Technology, 9 Institutskiy Lane, Dolgoprudny City, Moscow Region 141700, Russia; 4 Department of Geosciences, Center for Materials by Design, and Institute for Advanced Computational Science, Stony Brook University, Stony Brook, New York 11794, USA; 5 School of Materials Science, Northwestern Polytechnical University, Xi’an 710072, China

## Abstract

Using ab initio evolutionary simulations, we predict the existence of five novel stable Li-N compounds at pressures from 0 to 100 GPa (Li_13_N, Li_5_N, Li_3_N_2_, LiN_2_, and LiN_5_). Structures of these compounds contain isolated N atoms, N_2_ dimers, polyacetylene-like N chains and N_5_ rings, respectively. The structure of Li_13_N consists of Li atoms and Li_12_N icosahedra (with N atom in the center of the Li_12_ icosahedron) – such icosahedra are not described by Wade-Jemmis electron counting rules and are unique. Electronic structure of Li-N compounds is found to dramatically depend on composition and pressure, making this system ideal for studying metal-insulator transitions. For example, the sequence of lowest-enthalpy structures of LiN_3_ shows peculiar electronic structure changes with increasing pressure: metal-insulator-metal-insulator. This work also resolves the previous controversies of theory and experiment on Li_2_N_2_.

Li-N system contains two well-known compounds: lithium nitride (Li_3_N) and lithium azide (LiN_3_). Li_3_N has potential use as an electrolyte in Li-batteries and a hydrogen storage medium^1^,^2^,^3^,^4^. Extensive experimental and theoretical investigations show that Li_3_N undergoes a sequence of phase transitions with increasing pressure. At ambient conditions, X-ray diffraction identified in Li_3_N a mixture of two phases: α-Li_3_N (P6/mmm) and metastable β-Li_3_N (P6_3_/mmc); at about 0.5 GPa, α-Li_3_N fully transforms to β-Li_3_N; a new phase γ-Li_3_N (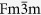) occurs near 40 GPa^5^,^6^. Normal ionic materials usually become metallic with increasing pressure, but Li_3_N is abnormal since the increasing pressure makes it into a much more strongly ionic state^7^. LiN_3_ has been widely used in industry as a nitrogen source, initial explosives and photographic materials^8^. Before 2013, LiN_3_ was known in a single phase C2/m, and seemed so simple and well understood. However, several other phases of LiN_3_ have been found using evolutionary crystal structure prediction methods coupled with first-principles calculations two years ago. At above 36 GPa, a hexagonal phase (P6/m) of LiN_3_ with pseudo-benzene N_6_ ring has been predicted by two research groups independently^9^,^10^. Some other phases appear as metastable: 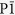 with a polyacetylene-like infinite linear nitrogen chain structure; C2/m and 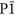 with puckered extended 2D decagonal and quasi-2D hexagonal nitrogen layers, respectively^10^. Above 375 GPa, Wang *et al.* identified the phase of P2_1_ which consists of zigzag N polymeric chains with N_5_ ring sharing N-N pairs^11^. The band structures indicate that there are two metal-insulator transitions in LiN_3_: first from insulator to metal at 36 GPa, and then from metal back to insulator at 375 GPa. Adding the new phases found in this work (see below), the sequence becomes metal-insulator-metal-insulator.

Besides nitrides (with N^3−^ anion) and azides ([N_3_]^−^), in 2001, Kniep *et al.* proved the existence of diazenides [N_2_]^2−^ by synthesizing SrN_2_ and BaN_2_ under high N_2_ pressure^12^,^13^. Since then, discovery of new diazenides has been of constant interest. In 2010, alkali diazenides Na_2_N_2_ and Li_2_N_2_ (Pmmm) were predicted^14^, but then, a different structure of Li_2_N_2_ (Immm) was obtained under HP/HT conditions (9 GPa, 750 K) by decomposition of LiN_3_^15^. This discrepancy encourages us to study Li_2_N_2_ under high pressure in detail.

Nitrogen can form many anionic species, e.g. [N_2_]^−^, [N_2_]^3−^ and [N_5_]^−^, which have just been obtained in molecular complexes^16^,^17^,^18^,^19^. We wonder if solid-state compounds with these anions in Li-N system could be synthesized under high pressure. Evolutionary algorithm USPEX has been widely used to predict new ground state structures in various systems without any experimental information, such as B-H, Xe-O, and Na-Cl^20^,^21^,^22^. The predicted counterintuitive compounds NaCl_3_ and Na_3_Cl in the Na-Cl system have been confirmed by experiment^22^. In this work, we have performed extensive structure searches on the Li-N system using variable-composition evolutionary algorithm USPEX, and indeed found many new stable compounds with very diverse and unusual crystal structures.

## Results and Discussion

**Figure 1:**
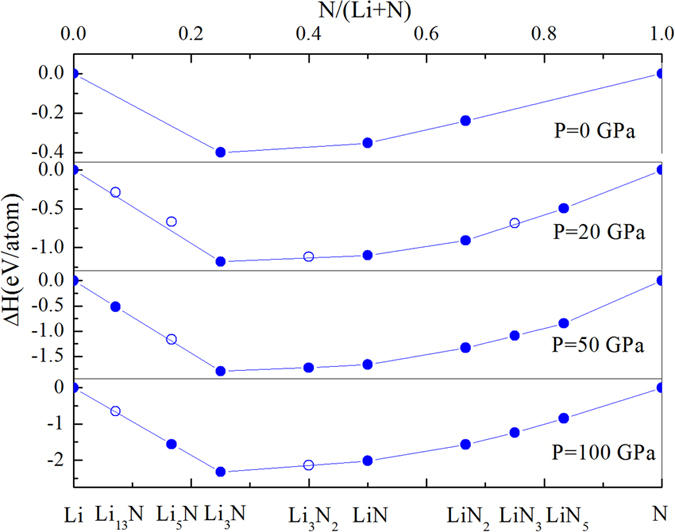
Convex hull diagrams for the Li-N system (showing enthalpies of formation (ΔH) of the compounds from ground-state Li and N) at 0, 20, 50, and 100 GPa.

We first studied the phase stability in the Li-N system by calculating the enthalpy of formation (ΔH) of Li-N compounds in the pressure range from 0 to 100 GPa. Stability of compounds is explored by the thermodynamic convex hull construction. If the enthalpy of decomposition of a compound into any other compounds is positive, then the compound is stable, which is depicted on the convex hull. The convex hulls are shown in Fig. 1 at selected pressures: 0, 20, 50, and 100 GPa. The various known phases of solid Li, N_2_, Li_3_N, LiN_3_, and Li_2_N_2_ are reproduced readily in our evolutionary structure searches. Interestingly, five previously unreported compositions of Li-N system: Li_13_N, Li_5_N, Li_3_N_2_, LiN_2_, and LiN_5_ are found to be on the convex hull under ambient or high pressure in our calculations. The calculated phonon spectra confirmed that all predicted structures are dynamically stable. In total, we have found three new N-rich compounds and two new Li-rich compounds.

Simultaneously with our work (in fact, with submission date after our paper appeared on arxiv.org) Peng *et al.*^23^ investigated the Li-N system and found two new stable compounds, LiN_2_ and LiN_5_. However, the phase diagram of the Li-N system published by Peng *et al.*^23^ missed a number of stable compounds (Li_13_N, Li_5_N, Li_3_N_2_). The enthalpies of reported phases in ref. 23 are recalculated and compared with our results. Detailed comparisons are shown in Figures S2 and S3 of the Supporting Information. Hence this paper presents a more complete and reliable picture, correcting omissions and presenting more stable crystal structures than those presented before.

We find that: (i) At ambient conditions (0 GPa), besides Li_3_N and Li_2_N_2_, LiN_2_ with space group P6_3_/mmc is surprisingly stable. These three compositions are always stable in the pressure range from 0 to 100 GPa. (ii) However, the long-known LiN_3_ is metastable below 49 GPa, which is in agreement with the known fact that it decomposes into N_2_ and Li under external influences (heat, irradiation, etc) at 0 GPa. (iii) At 20 GPa, LiN_5_ becomes stable, meanwhile Li_13_N, Li_3_N_2_ and LiN_3_ lie very close to (or nearly on) the convex hull. At 50 GPa, Li_13_N, Li_3_N_2_ and LiN_3_ are all stable, and Li_5_N lies very close to the convex hull. At 100 GPa, Li_5_N is stable, however, Li_13_N and Li_3_N_2_ are becoming metastable although they both lie nearly on the convex hull.

**Figure 2:**
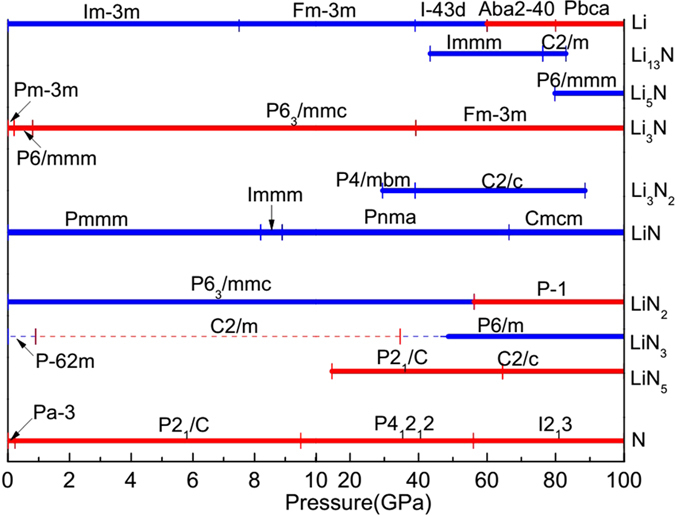
Pressure-composition phase diagram of the Li-N system from 0 to 100 GPa. The stable phases are shown in bold lines and the metastable phases of LiN_3_ are depicted in thin dash lines. Blue and red colors represent the metallic and insulating phases, respectively.

The pressure-composition phase diagram of the Li-N system is depicted in Fig. 2. For pure Li, with increasing pressure, the bcc phase (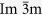) transforms into fcc (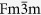), cI16 (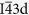), Aba2–40, and Pbca phases in sequence, which is in accordance with previous experimental and theoretical data^24^,^25^,^26^. For pure N, the known 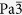, P2_1_/c, P4_1_2_1_2, and I2_1_3 structures are reproduced in our searches and agree well with other theoretical predictions^27^,^28^.

For Li_3_N there is a peculiar situation: the experimentally known at ambient conditions P6/mmm structure is predicted to be stable only at pressures above 0.2 GPa – at lower pressures, at the GGA level of theory, the 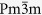 structure is more stable (at 0 GPa, by 22 meV/formula unit). This small upward shift of phase transition pressures is typical of the GGA, but one wonders whether 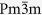 structure could be stabilized by impurities, temperature etc. The subsequent phases of Li_3_N in our calculations are all in agreement with the previous works^5^,^6^,^7^.

LiN_3_ is a thermodynamically stable compound (on the convex hull) only above 49 GPa, but it is well known also at ambient conditions as a metastable material. We found 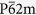 to have the lowest enthalpy in the pressure range 0–0.9 GPa, followed by C2/m and P6/m phases on increasing pressure. C2/m is the phase known experimentally at ambient conditions.

For LiN (actually Li_2_N_2_), the obtained structure at 0 GPa is Pmmm, which is consistent with the previous theoretical result^14^. At 8.2 GPa, Pmmm phase of LiN transforms into the Immm structure, indicating that the experimental result obtained at around 9 GPa is also perfectly correct^15^. At 8.9 GPa, the Immm structure will lose its stability and the Pnma phase becomes stable in the pressure range from 8.9 to 66.4 GPa. Then the Cmcm phase is stable up to 100 GPa.

Phase transformations of five new compositions of Li-N system are as follows: (i) For Li_13_N, the Immm structure is predicted to be stable from 43 to 76 GPa, following which the C2/m structure is stable up to 83 GPa. In fact, Immm and C2/m phases have nearly identical enthalpies (within 0.2 meV/atom), suggesting that Li_13_N can exist as a mixture of Immm and C2/m phases in the whole range of stability of this compound. (ii) Li_5_N has a single stable phase P6/mmm from 80 to at least 100 GPa. (iii) From 30 to 89 GPa, Li_3_N_2_ has two stable phases P4/mbm and C2/c. The pressure-induced structural transition from P4/mbm to C2/c occurs at about 39 GPa. (iv) Besides the P6_3_/mmc structure, LiN_2_ has another phase 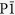, stable above 56 GPa, (v) For LiN_5_, the P2_1_/c structure becomes stable at 15 GPa and then transforms into the C2/c phase at the pressure of 65 GPa. The C2/c structure is stable at least up to 100 GPa.

**Figure 3:**
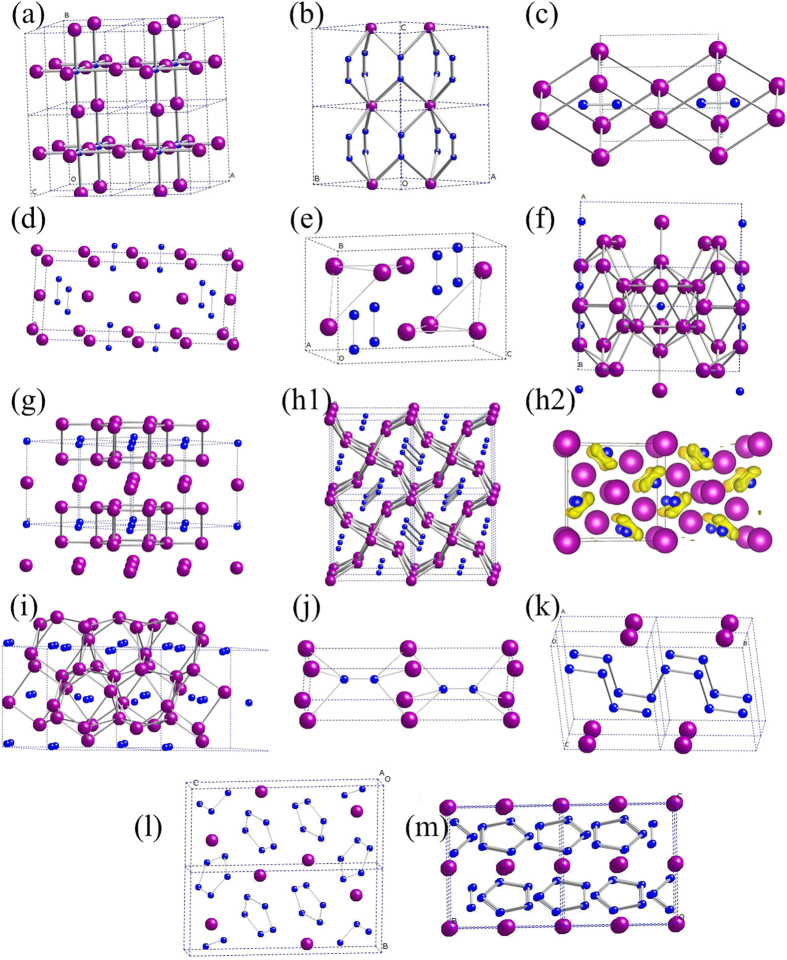
Crystal structures of Li-N compounds. (**a**) 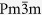-Li_3_N at 0 GPa, (**b**) 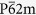 -LiN_3_ at 0 GPa, (**c**) Pmmm-Li_2_N_2_ at 0 GPa, (**d**) Immm-Li_2_N_2_ at 10 GPa, (**e**) Pnma-Li_2_N_2_ at 10 GPa, (**f**) Immm-Li_13_N at 50 GPa, (**g**) P6/mmm-Li_5_N at 90 GPa, (**h1**) P4/mbm-Li_3_N_2_ at 30 GPa, (**h2**) ELF isosurfaces (ELF = 0.85) of (**h1**), (i) C2/c-Li_3_N_2_ at 40 GPa, (**j**) P6_3_/mmc-LiN_2_ at 0 GPa, (**k**) 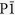-LiN_2_ at 60 GPa, (**l**) P2_1_/c-LiN_5_ at at 50 GPa, (**m**) C2/c-LiN_5_ at 80 GPa.

The representative structures of above-mentioned Li-N compounds under ambient conditions and high pressure are presented in Fig. 3. We first analyze the structures of Li_3_N and LiN_3_, and remind that the lengths of nitrogen-nitrogen bonds are 1.10 Å for the triple N–N bond, 1.25 Å for the double N = N bond, and 1.45 Å for the single N-N bond. (i) For Li_3_N, the calculated lattice parameters of P6/mmm, P6_3_/mmc and 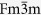 are in agreement with experimental data within 0.5%. The 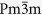 structure is very simple, an anti-ReO_3_-type structure made of corner-sharing NLi_6_ octahedra (Fig. 3a). Interestingly, across the phase transitions, the number of Li atoms surrounding each N atom increases from 6 for 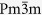 to 8 for P6/mmm, 11 for P6_3_/mmc and 14 for 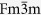. (ii) for LiN_3_ at ambient conditions, C2/m structure consists of Li^+^ cations and linear azide anions [N_3_]^−^ 10. As illustrated in Fig. 3b, unlike the C2/m structure, 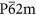 phase does have [N_3_]^−^ anions, but instead its unit cell contains two Li atoms and three N_2_ groups with the N-N distance of 1.151 Å at 0 GPa, smaller than that in the azide-ion [N_3_]^−^ (1.184 Å), but larger than that in the gas-phase N_2_ molecule (1.10 Å) and indicating a bond order between 2 and 3.

The Pmmm structure of Li_2_N_2_ consists of face-sharing Li_8_ parallelepipeds (Fig. 3c). The N_2_ groups sit in the center of parallelepipeds, which can be viewed that there are six Li atoms connecting to each N atom of N_2_ molecule and each of four Li atoms connects to both N atoms. The N-N bond length is 1.263 Å at 0 GPa, slightly larger than that of Na_2_N_2_ (1.24 Å)^14^ and indicating a double N = N bond and ideal charge of the N_2_ group equal to −2, which matches perfectly the formula Li_2_N_2_. Our calculated lattice constants of Immm structure (Fig. 3d) are in good agreement with experimental results^15^. The predicted N-N bond length is 1.271 Å, slightly smaller than the experiment. Figure 3e presents the Pnma structure of Li_2_N_2_ at 10 GPa. Its unit cell contains four N_2_^2−^ groups and eight Li^+^ ions. The N-N bond length is 1.269 Å.

For Li_13_N, Immm and C2/m phases have similar structures. The Immm structure of Li_13_N at 50 GPa is shown in Fig. 3f. This structure is an interesting example of Li-N compounds which can be viewed as a combination of a single Li atom and a slightly distorted Li_12_N icosahedral group (with N atom inside the Li_12_ icosahedron). A similar Li_12_Cs icosahedron is present in the Pnna structure of Li_3_Cs compound^29^, where neighboring icosahedra share Li-Li edges. However, the Li_12_N icosahedra are isolated and do not share Li atoms with each other in our Li_13_N compound. The Li-N bond lengths in the Li_12_N icosahedron are 1.934, 1.951 and 2.011 Å, i.e. nearly identical, and Li-Li distances are also nearly identical, ranging from 2.026 to 2.113 Å (maximum difference 4.3%, to compare with 22.3% in Li_3_Cs^29^).

The P6/mmm phase of Li_5_N has a layered structure, made of alternating layers of stoichiometry Li_4_N (here, N atoms are sandwiched between two Li-graphene sheets) and Li, see Fig. 3g. Such unusual layered structures with alternation of “metallic” and “non-metallic” layers have been previously reported by some of us for the Na-Cl system (e.g., Na_3_Cl, also confirmed experimentally^22^) and for the K-Cl system^30^. Bader analysis shows that Li_5_N at 90 GPa has charge configuration [Li_4_N]^–0.68^ Li^+0.68^, indicating that most of the valence electrons of Li layer transfer to the Li_4_N sandwich layer^31^. Interestingly, the Bader charge of Li atom in upper Li-graphene sheet of Li_4_N sandwich layer is nearly neutral (+0.1 e) and the charge of Li atom in bottom Li-graphene sheet is +0.74 e.

As observed in Fig. 3h1, the P4/mbm structure of Li_3_N_2_ consists of a three-dimensional network of Li atoms, which has open channels along z direction. This structure is very similar to the structure of the new compound Mg_3_O_2_ predicted by some of us recently^32^, except that in Li_3_N_2_ there is pairing of N atoms with the N-N distance of 1.353 Å at 30 GPa, indicating bond order between 1 and 2. Just like in P4/mbm-Mg_3_O_2_, we can clearly see columns of face-sharing body-centered cubes of metal atoms. The electron localization function (ELF) of Li_3_N_2_ (Fig. 3h2) shows strong charge transfer from Li to N. However, unlike Mg_3_O_2_ which is an electride, there is no strong interstitial electron location in Li_3_N_2_. Bader analysis also confirms the above result. The charges of P4/mbm-Li_3_N_2_ are +0.794 e for one Li atom, +0.809 e for the other two Li atoms, and −1.146e and −1.266 e for two N atoms, respectively. The C2/c structure has a more complex three-dimensional network of lithium atoms with N_2_ groups also sitting in its channels (Fig. 3i), with the N-N distance of 1.391 Å at 40 GPa.

The P6_3_/mmc structure of LiN_2_ can be described as a NiAs-type structure, where anionic positions are occupied by the N_2_ groups (Fig. 3j). At zero pressure, the N-N distance is 1.173 Å, indicating a bond order between 2 and 3. The 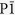 phase contains an infinite polyacetylene-like nitrogen chain (Fig. 3k), similar to the metastable phase of LiN_3_^10^. The N-N distances are 1.316, 1.320 and 1.333 Å at 60 GPa, suggesting bond order between 1 and 2. We can clearly see how pressure destroys molecular groups, favoring extended structures.

As observed in Fig. 3l, the P2_1_/c structure of LiN_5_ consists of isolated Li atoms and N_5_ rings, which up to now were only detected in molecular complexes^19^. At 50 GPa, the N-N distances are 1.286, 1.291, 1.299, 1.303 and 1.305 Å, respectively. The higher-pressure C2/c phase also consists of isolated Li atoms and N_5_ rings (Fig. 3m). Unlike in P2_1_/c, the N_5_ ring here is a nearly isosceles pentagon, with N-N distances of 1.277, 1.277, 1.301, 1.301, and 1.281 Å, respectively, at 80 GPa.

To obtain deeper insight into these new Li-N compounds, we calculated their band structures and density of states (DOS) at selected pressures. We found that all stable phases of Li_13_N, Li_5_N and Li_3_N_2_ are metallic. The 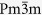 phase of Li_3_N is a semiconductor with the DFT band gap of 0.84 eV. All three stable phases of Li_2_N_2_ are also metallic, in agreement with experiment^15^. Interestingly, LiN_2_ has a metal-insulator transition: P6_3_/mmc is metallic at low pressure, but semiconducting in the high-pressure 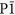 phase, with the band gap of 0.13 eV at 60 GPa. Since the newly predicted 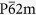 phase of LiN_3_ is also metallic, combining with previously known phases of LiN_3_, we find that the sequence of transitions of LiN_3_ under pressure is extremely unusual: from metallic to insulating to metallic to insulating. The P2_1_/c and C2/c phases of LiN_5_ are wide-gap insulators: e.g., the DFT band gap of the C2/c phase at 80 GPa is 2.19 eV.

**Figure 4:**
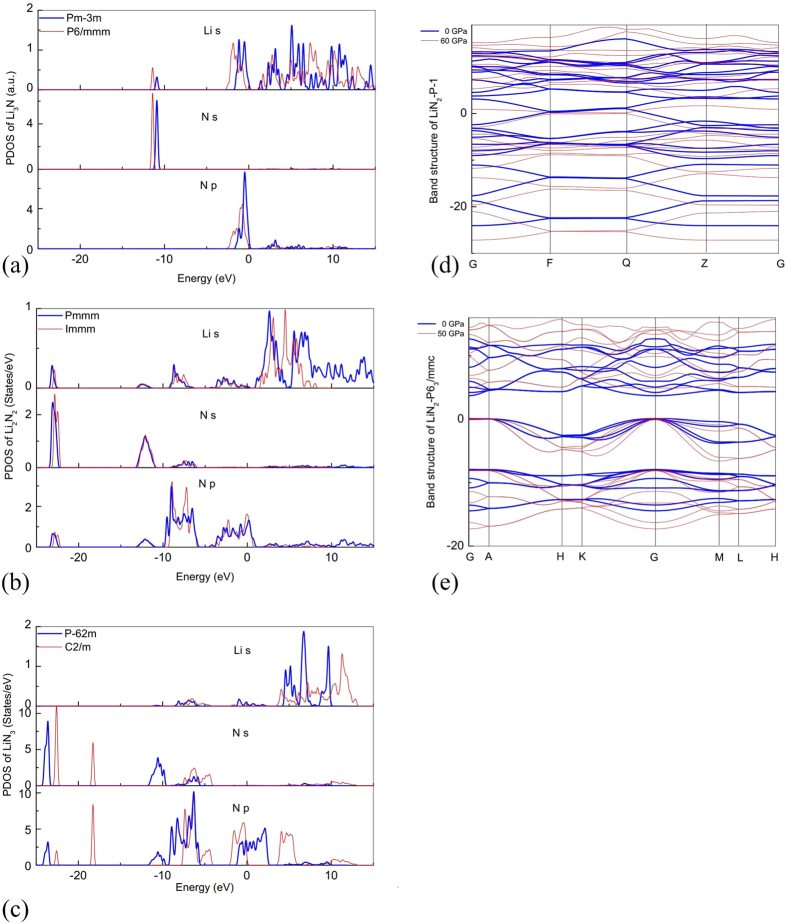
Electronic structures of selected Li-N compounds. (**a**–**c**) PDOSs of Li_3_N, Li_2_N_2_ and LiN_3_ at 0 GPa. (**d–e**) Band structures of 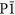 -LiN_2_ and P6_3_/mmc-LiN_2_ at two different pressures.

Some electronic structures are shown in Fig. 4. As is seen from Fig. 4(a–c), the PDOSs of two different phases of Li_3_N (or Li_2_N_2_ or LiN_3_) at 0 GPa are obviously different. The newly found phases 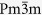 -Li_3_N, Pmmm-Li_2_N_2_ and 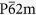-LiN_3_ have one character in common: the states near the Fermi level come mostly from Li-s and N-p orbitals. Figure 4(d–e) show the band structures of 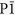-LiN_2_ and P6_3_/mmc-LiN_2_ at different pressures, respectively. The band structures at different pressures for the same phase are similar. When pressure increases, the band structure is more dispersive and the bandwidths also increase: both the conduction and valence bands broaden, and conduction band tends to shift upwards in energy. These changes can lead to both metallization and demetallization: for example, 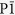-LiN_2_ is metallic at 0 GPa, whereas it becomes semiconductor with the gap of 0.13 eV at 60 GPa.

## Conclusions

A number of new Li-N compounds have been predicted using *ab initio* evolutionary structure search. Other than the well-known compositions Li_3_N, Li_2_N_2_ and LiN_3_, we found five novel compositions which should be experimentally synthesizable under pressure, including Li_13_N, Li_5_N, Li_3_N_2_, LiN_2_, and LiN_5_. Notably, the N-N bonding patterns evolve from isolated N ions to N_2_ dumbbells, to linear N_3_ groups, infinite nitrogen chains, N_5_ rings with increasing N content. Interestingly, for the experimentally known compounds Li_3_N and LiN_3_ at ambient conditions we find new lowest-energy structures (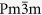 and 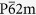, respectively): these are stable (at the GGA level of theory) in very narrow pressure ranges near 0 GPa. While this is most likely an artefact of the GGA (known to slightly overstabilize open structures and shift phase transition pressures upwards), these phases may be stabilized by doping, temperature, etc. We also resolve previous discrepancy on stable phases of Li_2_N_2_. In conclusion, this paper presents a more complete and reliable picture, correcting omissions and presenting more stable crystal structures than those presented before. Our work provides the basis for the future experimental investigations of the Li-N system.

## Methods

To search for stable compounds, the Li-N system was first explored using the variable-composition evolutionary technique, as implemented in the USPEX code^33^,^34^,^35^. Evolutionary crystal structure predictions were performed in the pressure range from 0 to 100 GPa. Initial structures included up to 16 atoms in the unit cell. The first generation of structures was produced randomly. The child structures were obtained applying heredity, transmutation, softmutation, and random symmetric generator, with probabilities of 40, 20, 20 and 20%, respectively. Then we performed detailed fixed-composition evolutionary calculations to explore the most promising compositions.

All structure relaxations and electronic structure calculations were done using the Vienna Ab Initio Simulation Package (VASP) in the framework of density functional theory^36^. The Perdew-Burke-Ernzerhof generalized gradient approximation (PBE-GGA) was employed to treat the exchange-correlation energy^37^, and the all-electron projector augmented wave (PAW) potentials were used to describe the core-valence interactions^38^. The cut-off energy of 650 eV and Monkhorst-Pack k-point meshes for sampling the Brillouin zone with resolution 2π × 0.04 Å^−1^ ensured that all the enthalpy calculations were well converged to better than 1 meV/atom. To ensure that the structures of predicted compounds in Li-N system are dynamically stable, phonon calculations were carried out using the Phonopy code^39^. Our tests showed that the effect of van der Waals interactions^40^,^41^ on stability of lithium nitrides is negligible, which is consistent with other works^23^.

## Additional Information

**How to cite this article**: Shen, Y. *et al.* Novel lithium-nitrogen compounds at ambient and high pressures. *Sci. Rep.* **5**, 14204; doi: 10.1038/srep14204 (2015).

## Supplementary Material

Supplementary Information
