# Supplementary material for: Novel lithium-nitrogen compounds at ambient and high pressures
Source: Sci Rep. 2015 Sep 16;5:14204. doi: 10.1038/srep14204 (PMC4570992; doi:10.1038/srep14204)
Supplement: Supplementary Information [file srep14204-s1.doc]

**Supplementary Materials**

**Novel lithium-nitrogen compounds at ambient and high pressures**

Yanqing Shen,^1,4,*^ Artem R. Oganov,^2,3,4,5^ Guangri Qian,^4^ Jin Zhang, ^4^ Huafeng Dong,^4^ Qiang Zhu,^4^ Zhongxiang Zhou^1^

1 Department of Physics, Harbin Institute of Technology, Harbin 150001, China

2 Skolkovo Institute of Science and Technology, Skolkovo Innovation Center, 3 Nobel St., Moscow 143026, Russia.

3 Moscow Institute of Physics and Technology, 9 Institutskiy Lane, Dolgoprudny City, Moscow Region 141700, Russia

4 Department of Geosciences, Center for Materials by Design, and Institute for Advanced Computational Science, Stony Brook University, Stony Brook, New York 11794, USA

5 School of Materials Science, Northwestern Polytechnical University, Xi’an 710072, China

* Corresponding author.

E-mail: shenyanqing2004@163.com

Phone number: 086045186414141

Fax number: 086045186414141

Figure.S1 **Enthalpies of predicted Li_3_N_2_ structures, relative to P4/mbm phase.**

Figure.S2 **Enthalpies of predicted Li_2_N_2_ structures, relative to Immm phase.** Peng *et al.* [1] found the phases P4_2_/mmc and I4/mmm and missed the phase Immm which is synthesized experimentally at 9 GPa [2]. As is shown, the phases P4_2_/mmc and I4/mmm (which are almost the same lines in the figure, shown in fine dash-dot lines) are less stable than the phases Immm [2] and Pnma found by us (shown in bold lines).

Figure.S3 **Enthalpies of predicted LiN_5_ structures, relative to P3_2_21 phase.** Peng *et al.* [1] found the phases P2_1_ and P2_1_/m of LiN_5_. As is shown, the phases P2_1_ and P2_1_/m (shown in fine dash lines) are less stable than the phases P2_1_/c and C2/c found by us (shown in bold lines).

Figure.S4 **Enthalpies of predicted LiN_3_ structures, relative to C2/m phase.**

Figure.S5 **Enthalpies of predicted LiN_2_ structures, relative to P6_3_/mmc phase.**

Figure.S6 **Enthalpies of predicted Li_5_N structures, relative to P6/mmm phase.**

Figure.S7 **Enthalpies of predicted Li_3_N structures, relative to P6/mmm phase.**

Fig.S8 **Enthalpies of predicted Li_13_N structures, relative to Immm phase.**


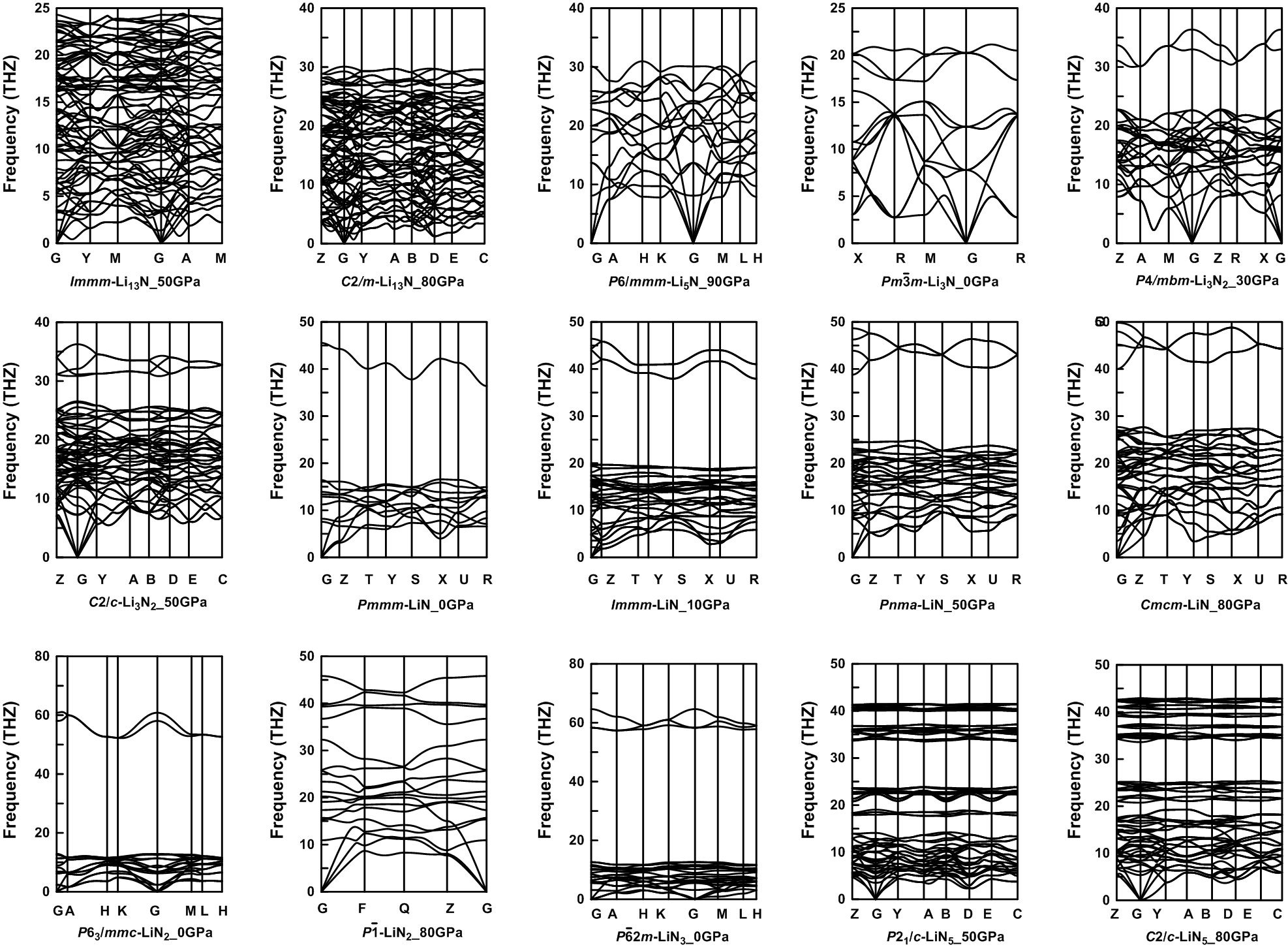


Fig. S9 **Phonon dispersion curves of Li-N compounds at selected pressures.**

**CIF files of Li-N compounds shown in Figure 3**

**Pm**
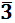
**m-Li_3_N at 0 GPa**

data_

_audit_creation_date

_audit_creation_method

_symmetry_space_group_name_H-M 'PM-3M'

_symmetry_Int_Tables_number 221

_symmetry_cell_setting cubic

loop_

_symmetry_equiv_pos_as_xyz

x,y,z

-x,-y,z

-x,y,-z

x,-y,-z

z,x,y

z,-x,-y

-z,-x,y

-z,x,-y

y,z,x

-y,z,-x

y,-z,-x

-y,-z,x

y,x,-z

-y,-x,-z

y,-x,z

-y,x,z

x,z,-y

-x,z,y

-x,-z,-y

x,-z,y

z,y,-x

z,-y,x

-z,y,x

-z,-y,-x

-x,-y,-z

x,y,-z

x,-y,z

-x,y,z

-z,-x,-y

-z,x,y

z,x,-y

z,-x,y

-y,-z,-x

y,-z,x

-y,z,x

y,z,-x

-y,-x,z

y,x,z

-y,x,-z

y,-x,-z

-x,-z,y

x,-z,-y

x,z,y

-x,z,-y

-z,-y,x

-z,y,-x

z,-y,-x

z,y,x

_cell_length_a 3.8729

_cell_length_b 3.8729

_cell_length_c 3.8729

_cell_angle_alpha 90.0000

_cell_angle_beta 90.0000

_cell_angle_gamma 90.0000

loop_

_atom_site_label

_atom_site_type_symbol

_atom_site_fract_x

_atom_site_fract_y

_atom_site_fract_z

_atom_site_U_iso_or_equiv

_atom_site_adp_type

_atom_site_occupancy

N1 N 0.50000 0.50000 0.50000 0.01267 Uiso 1.00

Li1 Li 0.50000 0.50000 0.00000 0.01267 Uiso 1.00

**P**
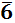
**2m-LiN_3_ at 0 GPa**

data

_audit_creation_date

_audit_creation_method

_symmetry_space_group_name_H-M 'P-62M'

_symmetry_Int_Tables_number 189

_symmetry_cell_setting hexagonal

loop_

_symmetry_equiv_pos_as_xyz

x,y,z

-y,x-y,z

-x+y,-x,z

x,y,-z

-y,x-y,-z

-x+y,-x,-z

y,x,-z

x-y,-y,-z

-x,-x+y,-z

y,x,z

x-y,-y,z

-x,-x+y,z

_cell_length_a 5.4288

_cell_length_b 5.4288

_cell_length_c 4.1925

_cell_angle_alpha 90.0000

_cell_angle_beta 90.0000

_cell_angle_gamma 120.0000

loop_

_atom_site_label

_atom_site_type_symbol

_atom_site_fract_x

_atom_site_fract_y

_atom_site_fract_z

_atom_site_U_iso_or_equiv

_atom_site_adp_type

_atom_site_occupancy

Li1 Li 0.66667 0.33333 0.00000 0.00000 Uiso 1.00

N1 N 0.40830 0.40830 0.63730 0.00000 Uiso 1.00

**Pmmm-Li_2_N_2_ at 0 GPa**

data

_audit_creation_date

_audit_creation_method

_symmetry_space_group_name_H-M 'PMMM'

_symmetry_Int_Tables_number 47

_symmetry_cell_setting orthorhombic

loop_

_symmetry_equiv_pos_as_xyz

x,y,z

-x,-y,z

-x,y,-z

x,-y,-z

-x,-y,-z

x,y,-z

x,-y,z

-x,y,z

_cell_length_a 2.7567

_cell_length_b 3.0486

_cell_length_c 4.3931

_cell_angle_alpha 90.0000

_cell_angle_beta 90.0000

_cell_angle_gamma 90.0000

loop_

_atom_site_label

_atom_site_type_symbol

_atom_site_fract_x

_atom_site_fract_y

_atom_site_fract_z

_atom_site_U_iso_or_equiv

_atom_site_adp_type

_atom_site_occupancy

Li1 Li 0.50000 0.00000 0.00000 0.00000 Uiso 1.00

Li2 Li 0.50000 0.50000 0.50000 0.00000 Uiso 1.00

N1 N 0.00000 0.50000 0.85620 0.00000 Uiso 1.00

**Immm-Li_2_N_2_ at 10 GPa,**

data

_audit_creation_date

_audit_creation_method '

_symmetry_space_group_name_H-M 'IMMM'

_symmetry_Int_Tables_number 71

_symmetry_cell_setting orthorhombic

loop_

_symmetry_equiv_pos_as_xyz

x,y,z

-x,-y,z

-x,y,-z

x,-y,-z

-x,-y,-z

x,y,-z

x,-y,z

-x,y,z

x+1/2,y+1/2,z+1/2

-x+1/2,-y+1/2,z+1/2

-x+1/2,y+1/2,-z+1/2

x+1/2,-y+1/2,-z+1/2

-x+1/2,-y+1/2,-z+1/2

x+1/2,y+1/2,-z+1/2

x+1/2,-y+1/2,z+1/2

-x+1/2,y+1/2,z+1/2

_cell_length_a 3.0882

_cell_length_b 4.4341

_cell_length_c 10.7127

_cell_angle_alpha 90.0000

_cell_angle_beta 90.0000

_cell_angle_gamma 90.0000

loop_

_atom_site_label

_atom_site_type_symbol

_atom_site_fract_x

_atom_site_fract_y

_atom_site_fract_z

_atom_site_U_iso_or_equiv

_atom_site_adp_type

_atom_site_occupancy

Li1 Li 0.00000 0.00000 0.00000 0.00000 Uiso 1.00

Li2 Li 0.50000 0.50000 -0.00000 0.00000 Uiso 1.00

Li3 Li 0.50000 0.50000 0.25230 0.00000 Uiso 1.00

N1 N 0.50000 0.14330 0.62290 0.00000 Uiso 1.00

**Pnma-Li_2_N_2_ at 10 GPa**

data

_audit_creation_date

_audit_creation_method ''

_symmetry_space_group_name_H-M 'PNMA'

_symmetry_Int_Tables_number 62

_symmetry_cell_setting orthorhombic

loop_

_symmetry_equiv_pos_as_xyz

x,y,z

-x+1/2,-y,z+1/2

-x,y+1/2,-z

x+1/2,-y+1/2,-z+1/2

-x,-y,-z

x+1/2,y,-z+1/2

x,-y+1/2,z

-x+1/2,y+1/2,z+1/2

_cell_length_a 4.7402

_cell_length_b 4.1109

_cell_length_c 6.2978

_cell_angle_alpha 90.0000

_cell_angle_beta 90.0000

_cell_angle_gamma 90.0000

loop_

_atom_site_label

_atom_site_type_symbol

_atom_site_fract_x

_atom_site_fract_y

_atom_site_fract_z

_atom_site_U_iso_or_equiv

_atom_site_adp_type

_atom_site_occupancy

N1 N -0.26460 0.09570 0.14100 0.00000 Uiso 1.00

Li1 Li 0.07500 0.25000 0.65480 0.00000 Uiso 1.00

Li2 Li 0.13240 0.25000 0.06790 0.00000 Uiso 1.00

**Immm-Li_13_N at 50 GPa,**

data

_audit_creation_date

_audit_creation_method ''

_symmetry_space_group_name_H-M 'IMMM'

_symmetry_Int_Tables_number 71

_symmetry_cell_setting orthorhombic

loop_

_symmetry_equiv_pos_as_xyz

x,y,z

-x,-y,z

-x,y,-z

x,-y,-z

-x,-y,-z

x,y,-z

x,-y,z

-x,y,z

x+1/2,y+1/2,z+1/2

-x+1/2,-y+1/2,z+1/2

-x+1/2,y+1/2,-z+1/2

x+1/2,-y+1/2,-z+1/2

-x+1/2,-y+1/2,-z+1/2

x+1/2,y+1/2,-z+1/2

x+1/2,-y+1/2,z+1/2

-x+1/2,y+1/2,z+1/2

_cell_length_a 7.0769

_cell_length_b 5.5707

_cell_length_c 5.2791

_cell_angle_alpha 90.0000

_cell_angle_beta 90.0000

_cell_angle_gamma 90.0000

loop_

_atom_site_label

_atom_site_type_symbol

_atom_site_fract_x

_atom_site_fract_y

_atom_site_fract_z

_atom_site_U_iso_or_equiv

_atom_site_adp_type

_atom_site_occupancy

Li1 Li 0.50000 0.81280 0.31310 0.00000 Uiso 1.00

Li10 Li 0.14390 0.18870 0.50000 0.00000 Uiso 1.00

Li17 Li 0.27020 -0.00000 0.19870 0.00000 Uiso 1.00

Li25 Li 0.50000 0.50000 0.50000 0.00000 Uiso 1.00

N1 N -0.00000 0.50000 0.50000 0.00000 Uiso 1.00

**P6/mmm-Li_5_N at 90 GPa**,

data

_audit_creation_date

_audit_creation_method ''

_symmetry_space_group_name_H-M 'P6/MMM'

_symmetry_Int_Tables_number 191

_symmetry_cell_setting hexagonal

loop_

_symmetry_equiv_pos_as_xyz

x,y,z

-y,x-y,z

-x+y,-x,z

-x,-y,z

y,-x+y,z

x-y,x,z

y,x,-z

x-y,-y,-z

-x,-x+y,-z

-y,-x,-z

-x+y,y,-z

x,x-y,-z

-x,-y,-z

y,-x+y,-z

x-y,x,-z

x,y,-z

-y,x-y,-z

-x+y,-x,-z

-y,-x,z

-x+y,y,z

x,x-y,z

y,x,z

x-y,-y,z

-x,-x+y,z

_cell_length_a 2.9867

_cell_length_b 2.9867

_cell_length_c 4.0842

_cell_angle_alpha 90.0000

_cell_angle_beta 90.0000

_cell_angle_gamma 120.0000

loop_

_atom_site_label

_atom_site_type_symbol

_atom_site_fract_x

_atom_site_fract_y

_atom_site_fract_z

_atom_site_U_iso_or_equiv

_atom_site_adp_type

_atom_site_occupancy

N1 N 0.00000 0.00000 0.00000 0.01267 Uiso 1.00

Li1 Li 0.66667 0.33333 0.21066 0.01267 Uiso 1.00

Li2 Li 0.00000 0.00000 0.50000 0.01267 Uiso 1.00

**P4/mbm-Li_3_N_2_ at 30 GPa**

data

_audit_creation_date

_audit_creation_method ''

_symmetry_space_group_name_H-M 'P4/MBM'

_symmetry_Int_Tables_number 127

_symmetry_cell_setting tetragonal

loop_

_symmetry_equiv_pos_as_xyz

x,y,z

-x,-y,z

-y,x,z

y,-x,z

-x+1/2,y+1/2,-z

x+1/2,-y+1/2,-z

y+1/2,x+1/2,-z

-y+1/2,-x+1/2,-z

-x,-y,-z

x,y,-z

y,-x,-z

-y,x,-z

x+1/2,-y+1/2,z

-x+1/2,y+1/2,z

-y+1/2,-x+1/2,z

y+1/2,x+1/2,z

_cell_length_a 4.7823

_cell_length_b 4.7823

_cell_length_c 2.7912

_cell_angle_alpha 90.0000

_cell_angle_beta 90.0000

_cell_angle_gamma 90.0000

loop_

_atom_site_label

_atom_site_type_symbol

_atom_site_fract_x

_atom_site_fract_y

_atom_site_fract_z

_atom_site_U_iso_or_equiv

_atom_site_adp_type

_atom_site_occupancy

Li1 Li 0.18110 0.68110 0.50000 0.01267 Uiso 1.00

Li2 Li 0.00000 0.00000 0.00000 0.01267 Uiso 1.00

N1 N 0.60000 0.10000 0.00000 0.01267 Uiso 1.00

**C2/c-Li_3_N_2_ at 40 GPa**

data

_audit_creation_date

_audit_creation_method '

_symmetry_space_group_name_H-M 'C2/C'

_symmetry_Int_Tables_number 15

_symmetry_cell_setting monoclinic

loop_

_symmetry_equiv_pos_as_xyz

x,y,z

-x,y,-z+1/2

-x,-y,-z

x,-y,z+1/2

x+1/2,y+1/2,z

-x+1/2,y+1/2,-z+1/2

-x+1/2,-y+1/2,-z

x+1/2,-y+1/2,z+1/2

_cell_length_a 4.9124

_cell_length_b 5.1516

_cell_length_c 5.8635

_cell_angle_alpha 90.0000

_cell_angle_beta 126.6481

_cell_angle_gamma 90.0000

loop_

_atom_site_label

_atom_site_type_symbol

_atom_site_fract_x

_atom_site_fract_y

_atom_site_fract_z

_atom_site_U_iso_or_equiv

_atom_site_adp_type

_atom_site_occupancy

Li1 Li -1.01700 -0.29270 -0.48390 0.00000 Uiso 1.00

N1 N 0.32360 -0.97780 0.16380 0.00000 Uiso 1.00

Li10 Li 0.00000 -0.93180 -0.25000 0.00000 Uiso 1.00

**P6_3_/mmc-LiN_2_ at 0 GPa**

data_

_audit_creation_date

_audit_creation_method ''

_symmetry_space_group_name_H-M 'P63/MMC'

_symmetry_Int_Tables_number 194

_symmetry_cell_setting hexagonal

loop_

_symmetry_equiv_pos_as_xyz

x,y,z

-y,x-y,z

-x+y,-x,z

-x,-y,z+1/2

y,-x+y,z+1/2

x-y,x,z+1/2

y,x,-z

x-y,-y,-z

-x,-x+y,-z

-y,-x,-z+1/2

-x+y,y,-z+1/2

x,x-y,-z+1/2

-x,-y,-z

y,-x+y,-z

x-y,x,-z

x,y,-z+1/2

-y,x-y,-z+1/2

-x+y,-x,-z+1/2

-y,-x,z

-x+y,y,z

x,x-y,z

y,x,z+1/2

x-y,-y,z+1/2

-x,-x+y,z+1/2

_cell_length_a 2.8916

_cell_length_b 2.8916

_cell_length_c 7.9716

_cell_angle_alpha 90.0000

_cell_angle_beta 90.0000

_cell_angle_gamma 120.0000

loop_

_atom_site_label

_atom_site_type_symbol

_atom_site_fract_x

_atom_site_fract_y

_atom_site_fract_z

_atom_site_U_iso_or_equiv

_atom_site_adp_type

_atom_site_occupancy

N1 N 0.66667 0.33333 0.67645 0.01267 Uiso 1.00

Li1 Li 0.00000 0.00000 0.50000 0.01267 Uiso 1.00

**P**
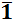
**-LiN_2_ at 60 GPa**

data

_audit_creation_date

_audit_creation_method '

_symmetry_space_group_name_H-M 'P-1'

_symmetry_Int_Tables_number 2

_symmetry_cell_setting triclinic

loop_

_symmetry_equiv_pos_as_xyz

x,y,z

-x,-y,-z

_cell_length_a 2.6457

_cell_length_b 3.7020

_cell_length_c 3.8525

_cell_angle_alpha 82.1197

_cell_angle_beta 100.3738

_cell_angle_gamma 70.4753

loop_

_atom_site_label

_atom_site_type_symbol

_atom_site_fract_x

_atom_site_fract_y

_atom_site_fract_z

_atom_site_U_iso_or_equiv

_atom_site_adp_type

_atom_site_occupancy

N1 N 0.48478 0.53446 0.66210 0.01267 Uiso 1.00

N2 N 0.15729 0.89794 0.65965 0.01267 Uiso 1.00

Li1 Li 0.82289 0.76614 0.02733 0.01267 Uiso 1.00

**P2_1_/c-LiN_5_ at at 50 GPa,**

data

_audit_creation_date

_audit_creation_method ''

_symmetry_space_group_name_H-M 'P21/C'

_symmetry_Int_Tables_number 14

_symmetry_cell_setting monoclinic

loop_

_symmetry_equiv_pos_as_xyz

x,y,z

-x,y+1/2,-z+1/2

-x,-y,-z

x,-y+1/2,z+1/2

_cell_length_a 3.9895

_cell_length_b 4.1537

_cell_length_c 10.1965

_cell_angle_alpha 90.0000

_cell_angle_beta 107.0485

_cell_angle_gamma 90.0000

loop_

_atom_site_label

_atom_site_type_symbol

_atom_site_fract_x

_atom_site_fract_y

_atom_site_fract_z

_atom_site_U_iso_or_equiv

_atom_site_adp_type

_atom_site_occupancy

Li1 Li -0.96480 0.62380 -0.10950 0.00000 Uiso 1.00

N1 N -1.33940 0.79970 -0.38690 0.00000 Uiso 1.00

N2 N 0.66860 0.22870 0.94000 0.00000 Uiso 1.00

N3 N 0.28650 0.44100 0.60350 0.00000 Uiso 1.00

N4 N -1.18990 0.57010 -0.30540 0.00000 Uiso 1.00

N5 N 0.42250 0.84510 0.81290 0.00000 Uiso 1.00

**C2/c-LiN_5_ at 80 GPa**

data

_audit_creation_date

_audit_creation_method '

_symmetry_space_group_name_H-M 'C2/C'

_symmetry_Int_Tables_number 15

_symmetry_cell_setting monoclinic

loop_

_symmetry_equiv_pos_as_xyz

x,y,z

-x,y,-z+1/2

-x,-y,-z

x,-y,z+1/2

x+1/2,y+1/2,z

-x+1/2,y+1/2,-z+1/2

-x+1/2,-y+1/2,-z

x+1/2,-y+1/2,z+1/2

_cell_length_a 5.8480

_cell_length_b 4.8002

_cell_length_c 5.2296

_cell_angle_alpha 90.0000

_cell_angle_beta 102.4636

_cell_angle_gamma 90.0000

loop_

_atom_site_label

_atom_site_type_symbol

_atom_site_fract_x

_atom_site_fract_y

_atom_site_fract_z

_atom_site_U_iso_or_equiv

_atom_site_adp_type

_atom_site_occupancy

N1 N 0.66670 0.55280 0.37620 0.00000 Uiso 1.00

N2 N 0.90050 0.19890 0.66960 0.00000 Uiso 1.00

Li1 Li 0.50000 0.00000 0.50000 0.00000 Uiso 1.00

N3 N 0.50000 0.71210 0.25000 0.00000 Uiso 1.00

Supplementary References:

[1] Peng, F., Yao, Y., Liu, H. & Ma, Y. Crystalline LiN_5_ predicted from first-principles as a possible high-energy material. J. Phys. Chem. Lett. **6,** 2363-2366 (2015).

[2] Schneider, S. B., Frankovsky, R. & Schnick, W. High-pressure synthesis and characterization of the alkali diazenide Li_2_N_2_. Angew. Chem. Int. Ed. **51,** 1873-1875 (2012).
